# Effects of UV-C Disinfection on N95 and KN95 Filtering Facepiece Respirator Reuse

**DOI:** 10.1128/aem.01221-22

**Published:** 2022-09-21

**Authors:** Castine Bernardy, Nicola Elardo, Alexa Trautz, Jim Malley, Diyuan Wang, Joel Ducoste

**Affiliations:** a Department of Civil and Environmental Engineering, University of New Hampshire, Durham, New Hampshire, USA; b Department of Civil, Construction, and Environmental Engineering, North Carolina State University, Raleigh, North Carolina, USA; Centers for Disease Control and Prevention

**Keywords:** COVID-19, FFRs, UV_254_, dose-response curve

## Abstract

The objective of this study was to evaluate the effectiveness of UV technology for virus disinfection to allow FFR reuse. UV is a proven decontamination tool for microbial pathogens, including the SARS-CoV-2 virus. Research findings suggest that the impacts of UV-C treatment on FFR material degradation should be confirmed using microbial surrogates in addition to the commonly performed abiotic particle testing. This study used the surrogates, E. coli and MS-2 bacteriophage, as they bracket the UV response of SARS-CoV-2. Lower log inactivation was observed on FFRs than predicted by aqueous-based UV dose-response data for MS-2 bacteriophage and E. coli. In addition, the dose-response curves did not follow the trends commonly observed with aqueous data for E. coli and MS-2. The dose-response curves for the respirators in this study had a semicircle shape, where the inactivation reached a peak and then decreased. This decrease in UV inactivation is thought to be due to the degradation of the fibers of the FFR and allows for more viral and bacterial cells to wash through the layers of the respirator. This degradation phenomenon was observed at UV doses at and above 2,000 mJ/cm^2^. Results have demonstrated that FFR materials yield various results in terms of effective disinfection in experiments conducted on KN95 and N95 face respirators. The highest inactivation for both surrogates was observed with the KN95 respirator made by Purism, yielding 3 and 2.75 log inactivation for E. coli and MS-2 at UV doses of 1,500 mJ/cm^2^. The KN95 made by Anboruo yielded the lowest inactivation for MS-2 at 0.75 log when exposed to 1,000 mJ/cm^2^. To further test the degradation theory, experiments used a collimated beam device to test the hypothesis further that degradation is occurring at and above UV doses of 1,500 mJ/cm^2^. The experiment aimed to determine the effect of “predosing” a respirator with UV before inoculating the respirator with MS-2. In this test, quantification of the penetrated irradiance value and the ability of each layer to retain MS-2 were quantified. The results of the experiments varied from the intact FFR degradation experiments but displayed some data to support the degradation theory.

**IMPORTANCE** Research suggests degradation of FFR materials at high UV doses is important. There appears to be a peak inactivation dose at approximately 1,500 mJ/cm^2^. The subsequent dose increases appear to have the reverse effect on inactivation values; these trends have shown true with both the N95 and KN95-Purism respirators.

## INTRODUCTION

The COVID-19 pandemic had a remarkable effect on public health globally. The viral pathogen responsible for the onset of the coronavirus disease, SARS-CoV-2, is an airborne pathogen spread by droplets emitted by breathing, coughing, sneezing, and speaking. Due to the ability of the viral particles to remain airborne, filtering facepiece respirators (FFRs) were utilized worldwide as personal protective equipment (PPE) to reduce the viral load the public was exposed to.

As COVID-19 hospitalizations increased and mask mandates took effect, a respirator shortage plagued the globe. First responders and frontline medical workers across the nation began reaching out to UV researchers requesting information about using UV (UV) to disinfect their used N95 and KN95 respirators for reuse. The CDC recommended using decontamination techniques such as UV disinfection in times of “crisis capacity” of available FFRs as a means of limiting self-contamination (Centers for Disease Control and Prevention [CDC], 2021) The objective of this study was to determine the efficacy of a commercial UV device (UV Lumin) as a disinfection technology to allow for the reuse of N95 and KN95 respirators in times of a shortage crises.

KN95 and N95 face respirators vary greatly in material type, number of layers, porosity, and structural design. N95 respirators filter at least 95% of 0.3 μm particles and are approved by the U.S. National Institute for Occupational Safety (NIOSH). Due to the COVID-19 pandemic, significant shortages of N95s existed worldwide; therefore, the standard Chinese respirator (KN95) was utilized by many when access to N95 respirators was limited. KN95 respirators do not have NIOSH approval and are less desirable for protection against aerosols/droplets as they have reduced filtration capacity compared to N95. Filtration efficiency for KN95 respirators has been reported between 53 and 85% (1).

N95 FFRs comprise three layers: filter, outer, and inner layers. The layers' materials consist of polyester, polypropylene, nylon, and cotton. Nonwoven polypropylene is a very common filter fabric material. The fibers of the nonwoven polypropylene fabric are random in arrangement, increasing the efficiency to trap droplets or particles (2).

Nonwoven polypropylene filters are either spun-bound or melt-blown during manufacturing. Spun-bound layers are generally used as the inner/outer layers of the respirator for structural integrity, as the fiber diameter is larger than that of the melt-blown fibers. The melt-blown layers of the respirator are responsible for the filtration efficiency of the respirator, as they have a significantly higher surface area than the spun-bound layers, 2 m^2^/g versus 0.2 m^2^/g, respectively. In addition, the melt-blown layer is treated with an electrostatic field during manufacturing to create an electrostatic charge with an affinity to trap small viral-sized particles inside the respirator. As the filter layer of the respirator significantly contributes to respirator filtration efficiency, it is generally 3 times thicker than the inner and outer layers of the respirator (2).

There are significant differences between the composition of N95 and KN95 respirators. It is common for the KN95 respirator to have four layers: filter, cotton, outer, and inner layers. The cotton layer of the respirator comprises 70% of the total respirator thickness. Unlike the N95 filter, the filter layer of the KN95 is the thinnest portion of the respirator, comprising less than 20% of the total thickness. Although the filter is significantly thinner than that of the N95, it serves the same purpose: it is the respirator layer where the droplets and particles are trapped. The filter dipole charge density is 8 times higher than the respirator's inner, outer, and cotton layers. In addition, the filter layer's porosity was reduced compared to other layers. The filter layer of the respirators is responsible for 90% of the filtration efficiency for both KN95 and N95 respirators (2).

## RESULTS

### Respirator types.

**FIG 1:**
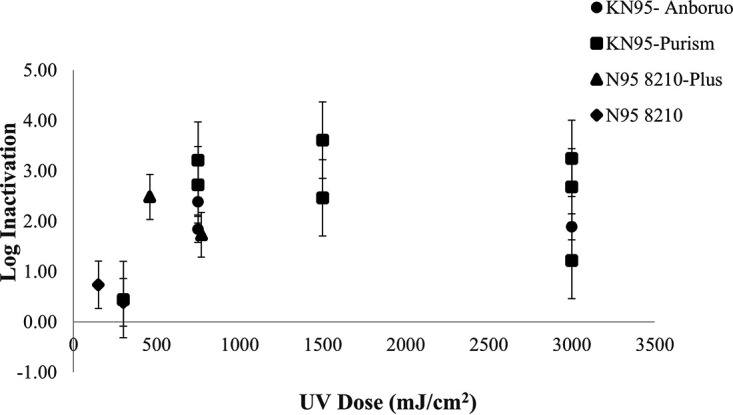
The UV dose-response curve for E. coli for the KN95-Anboruo, KN95-Pursim, N95 8210-Plus, and N95 8210 face respirators. The error bars displayed on the figure display the standard deviation of E. coli recovery tabulated from all control runs. These data can be seen in Table S2.

**FIG 2:**
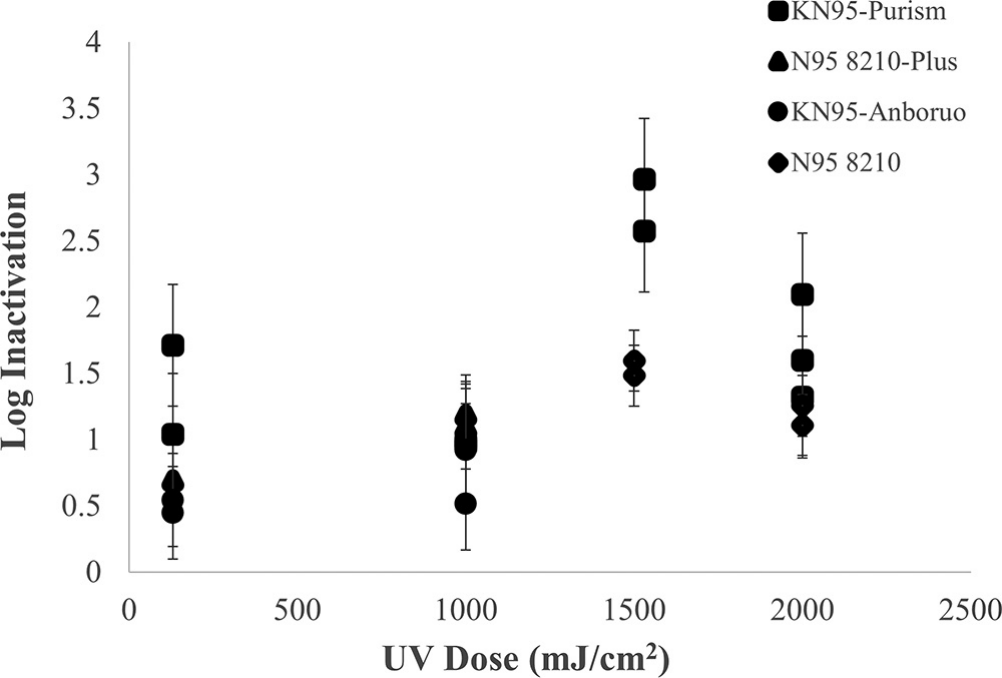
The UV dose-response curve for MS-2 bacteriophage for the KN95-Anboruo, KN95-Purism, N95 8210-Plus, and N95 8210 face respirators. The error bars displayed on the figure display the standard deviation of MS-2 bacteriophage recovery tabulated from all control runs. These data can be seen in Table S3.

UV disinfection using two surrogates (E. coli and MS-2 bacteriophage) was studied on four FFRs: KN95-Anboruo, KN95-Purism, and N95 8210-Plus and N95 8210. UV dose-response curves for both surrogates were created for all respirator types tested and are displayed in Fig. 1 and 2. Fig. 1 displays the log inactivation of E. coli on KN95-Anboruo, KN95-Purism, N95 8210-Plus, and N95 8210 respirators when exposed to UV doses ranging from 150 to 3,000 mJ/cm^2^. The KN95-Purism respirator achieved the highest inactivation values, followed by N95 8210-Plus and KN95-Anboruo, where N95 8210 displays the lowest inactivation. Two trials were run for each experiment, and the microbial variation (error bars) between the experiments can be seen by the spread of the data in the figure.

The N95 8210 experimentation was halted as the respirator type yielded poor disinfection results, as displayed in Fig. 1. The control runs demonstrated a high affinity for the bacterial cells, as very little E. coli was recovered during extraction. The decrease in the log inactivation as UV dose increased for N95 respirators tested was an unexpected trend observed in several experiments. Although this finding is counterintuitive to what one would expect to see in a UV dose-response curve, it is likely an artifact of microbial variation. Microbial variation in these experiments was up to +/− 1 log; therefore, the decreases in inactivation observed more likely demonstrates a plateau effect. More experiments would need to be completed to confirm the hypothesis that the N95 8210-Plus likely cannot achieve higher UV inactivation than 2 logs. The UV log inactivation in the N95 8210 could not be determined due to the high retention of E. coli in the controls as shown in a subsequent figure. In addition, the shape of the KN95-Purism curve is notable, as it sharply increased from doses 300 to 750 mJ/cm^2^ and then decreased between 1,500 and 3,000 mJ/cm^2^. This decreased inactivation cannot be explained simply by microbial variation and may suggest degradation of the respirator materials at the higher UV doses. The average retention of E. coli on KN95-Purism (5 trials) was 1.32 log as shown in a subsequent figure. It is hypothesized that as the respirator degrades, the E. coli retained within the fibers could then be recovered from the respirator. This observation indicates that for the KN95-Purism, UV doses higher than 1,500 mJ/cm^2^ may cause degradation of the respirator materials.

Similarly, the KN95-Anboruo respirator UV dose-response plateaued at 2 log inactivation. This effect is likely due to respirator degradation or microbial variation. This degradation of the electrostatic properties of the respirator materials at higher UV doses will be explored further in later experiments. The data suggest that KN95-Purism is the most suitable for UV disinfection for E. coli, as it achieves the highest UV inactivation (approximately 3 log) compared to the other respirators. Although, these data also suggest UV doses greater than 1,500 mJ/cm^2^ should not be used to avoid respirator degradation.

Figure 2 displays the log inactivation of MS-2 bacteriophage on KN95-Anboruo, KN95-Purism, N95 8210-Plus, and N95 8210 respirators. This figure displays data from UV doses ranging from 0 to 2,000 mJ/cm^2^. The figure suggests that the greatest MS-2 inactivation can be achieved with KN95-Purism followed by N95 8210. The peak inactivation for the KN95-Purism and N95 8210 respirators are approximately 2.75 and 1.5 log, respectively. The KN95-Anaboruo respirator behaves similarly for MS-2 as it did for E. coli in that the maximum UV inactivation of the surrogate plateaus.

### Controls.

**FIG 3:**
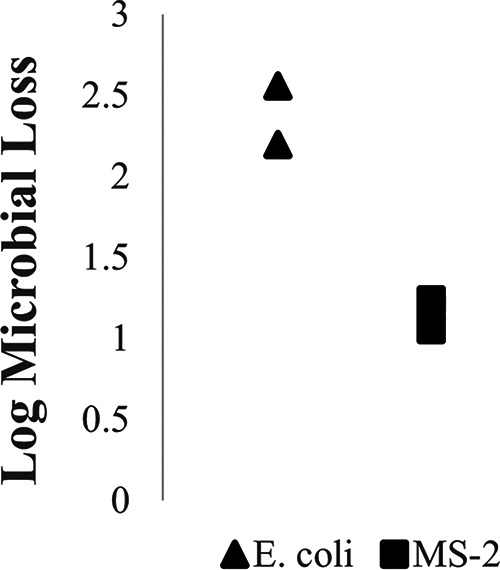
The log microbial loss of E. coli and MS-2 bacteriophage through the nebulizer. The log loss of *E. coli* is significantly higher than the log loss of MS-2 bacteriophage. This can be seen in the ANOVA table displayed in Fig. S4.

**FIG 4:**
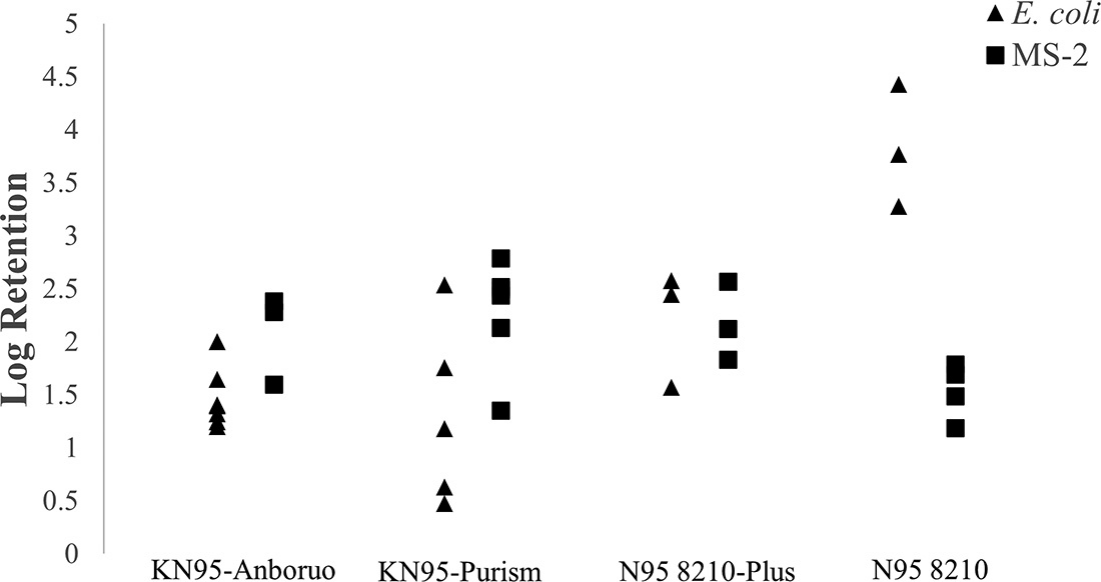
The log retention of E. coli and MS-2 bacteriophage of each mask type used for experimentation. These data display the results of the control runs, where a UV dose was not applied. These data were put into an ANOVA table to prove there exists a significant statistical difference between the FFRs retention capacity, which is shown in Fig. S1 and S2. The standard deviations of these data points were used to create the error bars shown in Fig. 1 and 2, the raw data are displayed in Tables S2 and S3.

It is equally important to evaluate control runs when determining UV log inactivation of organisms on face respirators. There are many sources of loss when determining the log loss of surrogates applied to a face respirator. Extraneous sources of loss include loss due to application process (nebulizer or dropper), desiccation, and retention of the surrogate in the internal fibers of the respirator. Fig. 3 displays the log loss of E. coli and MS-2 through the nebulizer. The nebulizer increases the pressure of its liquid reservoir and creates an aerosolized effluent spray. The loss of E. coli and MS-2 bacteriophage through the nebulizer are statistically different, which can be seen in the supplemental information section. E. coli has approximately twice the loss as MS-2 due to the nebulizer. Fig. 4 displays a significant variation in log retention of both experimental surrogates by respirator type. Each data point represents a different respirator. The retention of E. coli and MS-2 for the KN95-Anboruo and N95 8210-Plus respirator types do not vary significantly. The variation between E. coli and MS-2 retention for N95 8210 was quite large due to the difference in electrostatics of the respirator material.

Figure 4 also revealed that KN95-Purism had the most respirator-to-respirator variation in log retention values. The E. coli and MS-2 retention values varied from 0.47 to 2.5 log and 1.4 to 4.2 log, respectively. KN95-Purism has 5 layers, whereas the N95 respirator types have 3. There may be more retention variation in respirators that have more respirator layers, as the ability for the surrogate to be washed out depends upon what layer it was retained on when the aerosol was applied.

### Respirator degradation experiment.

Research suggests degradation of FFR materials at high UV doses is important. There appears to be a peak inactivation dose at approximately 1,500 mJ/cm^2^, and subsequent dose increases appear to have the reverse effect on inactivation values. These trends have shown true with both the N95 and KN95-Purism respirators. Therefore, to further test the hypothesis that degradation is occurring at and above UV doses of 1500 mJ/cm^2^, a degradation experiment was conducted. The goal of the experiment was to determine the effect of ‘predosing’ a respirator with UV prior to inoculating the respirator with MS-2.

**FIG 5:**
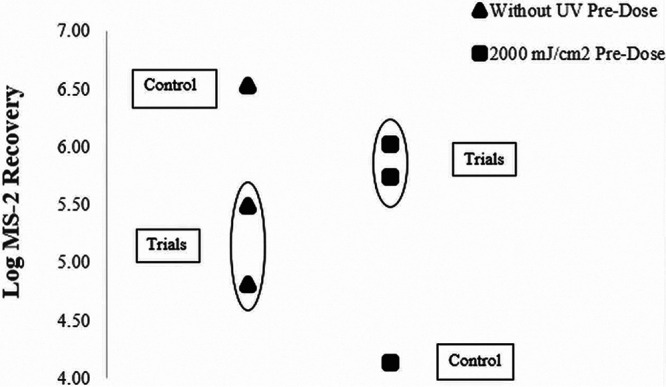
The results of “predosing” a KN95-Purism respirator with 2,000 mJ/cm^2^ prior to inoculating the respirator with MS-2 bacteriophage and exposing it to UV light. The results are compared to data that did not receive UV exposure before inoculation. This experiment tested a UV disinfection dose of 150 mJ/cm^2^; therefore, the “predosing” experiment incorporated this dose after the 2,000 mJ/cm^2^ predose, which tested FFR degradation. The “without UV predose” (triangle) control represents an experiment where the respirator was inoculated with MS-2 bacteriophage and then washed with sterile phosphate buffer solution (no UV dose). The “2,000 J/cm^2^ UV predose” (square) control represents an experiment where the respirator was inoculated with MS-2 bacteriophage, placed in the UV Lumin, where it received a dose of 150 mJ/cm^2^, and was washed wish sterile PBS. Unlike the (square) trial experiments, this control value did not receive the 2,000 J/cm^2^ predose.

Figure 5 compares experiments in which one data set (square data points) received a 2,000 mJ/cm^2^ dose before being inoculated with MS-2. The triangular data points were not pretreated with UV before experimentation. The square data points were collected for respirators pretreated with a 2,000 mJ/cm^2^ dose. These data suggest that approximately 1.8 logs more MS-2 were extracted off the respirator after receiving the high UV preexposure. When UV respirators receive UV doses of 2,000 mJ/cm^2^ or above, it reduces their ability to retain viral particles. This phenomenon is thought to be due to the degradation of the respirator materials.

**FIG 6:**
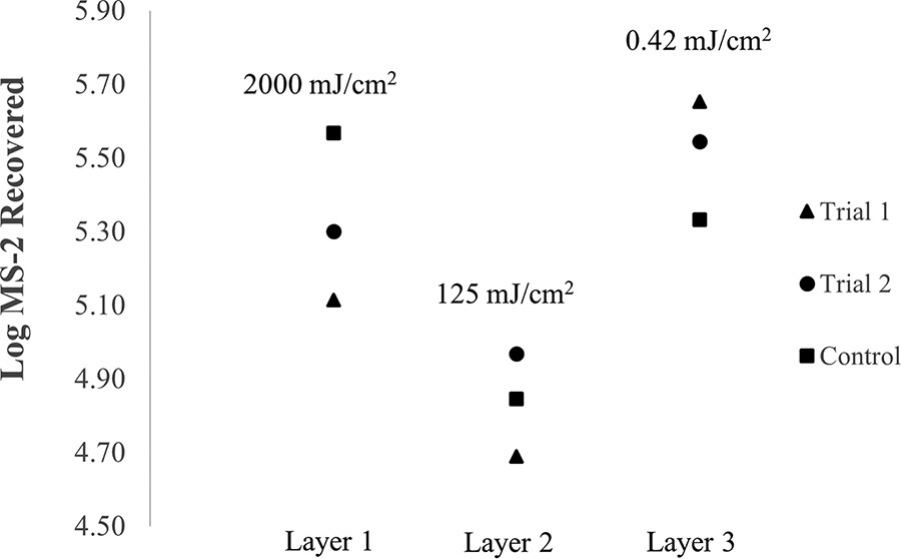
The results of a degradation experiment conducted using the collimated beam. An N95 8210 FFR was cut into coupons and the layers were separated to determine the irradiance penetration and the effect of degradation through the layers. Two trials were conducted for each layer, the control was not exposed to UV. Layers 1, 2 and 3 are comprised of polyester, polypropylene, and polyester, respectively.

**FIG 7:**
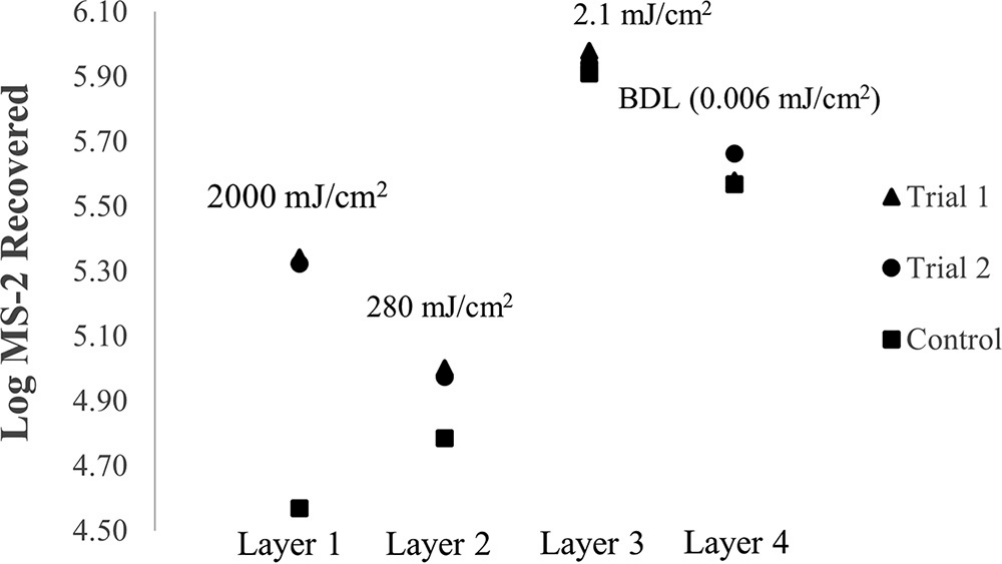
The results of a degradation experiment conducted using the collimated beam. A KN95-Purism FFR was cut into coupons, and the layers were separated to determine the irradiance penetration and the effect of degradation through the layers. Two trials were conducted for each layer; the control was not exposed to UV. Layers 1, 2, 3, and 4 are comprised of polypropylene spun-bound nonwoven fabric, polypropylene melt-blown nonwoven fabric, polyethylene-polyethylene terephthalate air-blown nonwoven fabric, and polypropylene spun-bound nonwoven fabric, respectively.

Figure 6 and 7 display the retention of MS-2 viral particles after UV treatment on 25 cm^2^ coupons of N95 and KN95-Purism, respectively. The top layer of the FFR received the target UV dose thought to be the cause of degradation, 2,000 mJ/cm^2^. The average irradiance used at the center point of the coupon was then used to determine the time the top layer needed to be exposed to yield a dose of 2,000 mJ/cm^2^. The penetrated irradiance value was multiplied by the exposure time to determine the subsequent doses that the lower layers received, as seen in Fig. 6. The layers were separated and preexposed to UV at the specified dose prior to MS-2 inoculation. The control coupons were not preexposed to UV.

The data displayed in Fig. 6 suggests that layer 1 does not show degradation (control recovery greater than treated recovery); layer 2 is inconclusive, and layer 3 showed a small degradation effect compared to the variability of the two trial runs to the control. Fig. 7 displays the results of the degradation experiment through the FFR layers using a KN95-Purism. The results of this experiment indicate a significant effect on layers 1 and 2 when comparing the reproducibility of trial 1 and 2, with the results of the control. Layers 3 and 4 showed the correct trend if degradation were occurring but cannot be considered a significant difference in log MS-2 recovery.

## DISCUSSION

### Dose response curves.

The difference in N95 behavior between MS-2 and E. coli is likely due to electrostatic interactions, between the respirator materials and the surrogates. The two respirator models are very similar. The outer material of the respirator (cover-web) comprises polypropylene and polyester for the N95 8210-Plus and N95 8210, respectively (3). When comparing the results of Fig. 1 and 2, the retention values of N95 8210-Plus and N95 8210 are more variable for E. coli than MS-2 bacteriophage. These results indicate that the retention on the respirators is less dependent on the size of the E. coli (1 to 2 μm [4]) or MS-2 (27 nm [5]), but rather the surface interactions occurring within the respirator since polyester is more hydrophilic than polypropylene (6). The respirators behaved similarly when MS-2 was utilized as a surrogate, as the data points followed the same trend as KN95-Purism, increasing until 1500 mJ/cm^2^, followed by decreased log inactivation.

Interestingly, the KN95-Purism behaved similarly with both surrogates. The inactivation values peak at 1500 mJ/cm^2^ for MS-2 and E. coli, reaching 2.75 and 3.0 log, respectively. The same pattern occurred with the N95 8210 respirator in Fig. 2, as the highest inactivation observed was approximately 1.5 log at a UV dose of 1500 mJ/cm^2^. The consistency of the log inactivation peaking at 1500 mJ/cm^2^ with both surrogates and with two respirator types further supports the hypothesis of respirator degradation at higher UV doses.

UV disinfection of respirators is challenging because the surrogate can be shielded by the respirator's pore spaces and layers, which is not a contributing factor for water UV dose-response curves. When the respirator was placed in the Lumin device, likely, UV light never reached some of the surrogates applied, therefore not inactivating E. coli or MS-2. This phenomenon has been labeled the “Canyon wall effect” (7) as the pore spaces between fibers of the outer layer are larger than the surrogate particle.

A study done by Fisher et al. revealed that the UV penetration of the layers varies by respirator type. In the study, six N95 FFRs were taken apart by layers. The UV light penetration through the exterior layer ranged from 23% to 50%, whereas the penetration through the filtering layers ranged from 0.05% to 22%. These findings suggest that the E. coli or MS-2 in the filtering layers of the respirator receive minimal UV irradiance. It also should be noted that the range of UV penetration is highly variable by respirator type (8). Lindsley et al. also noted these observations, who tested particle penetration of four N95 respirators. The research group studied NaCl particle penetration on four respirator types and found that penetration generally increased with UV dose and varied significantly between respirator types.

When considering the materials of many of these respirators, it is apparent that the some of the layers are damaged after significant exposure to UV_254_ light. As shown in Fig. 4, the materials of the respirator retain the surrogate within the internal fibers of the respirator. It is thought that as the respirator fibers degrade, they lose their ability to retain surrogate particles. Therefore, the surrogates are washed out of the respirator, indicating lower inactivation levels.

Within the literature, this concept of respirator degradation has been studied recently. In one study conducted on 254 and 265 nm wavelengths, researchers found no respirator degradation up to 10,000 mJ/cm^2^ (9). However, it should be noted that this study did not use microbial data to quantify their results. Instead, 50 to 220 nm silica particles were used to quantify filtration removal efficiency, and 100 nm polystyrene latex spheres to quantify capture efficiency. Tensile strength and pressure drop measurements were also recorded, and there were no alterations to the respirator integrity recorded (9).

A study conducted by Lindsley et al. tested higher UV doses (120 to 950 J/cm^2^) and observed degradation at the higher doses. The study tested flow penetration and flow resistance with NaCl particles ranging from 0.02 to 0.4 μm in diameter. The strength of the respirator coupons and straps were quantified using an ASTM bursting strength procedure and by forcing a 12.7 mm steel ball through the layers and recording the maximum force applied before failure. The mean flow resistance and penetration effects of the UV doses varied by respirator and dose (120 to 950 J/cm^2^). The flow resistance and penetration values before and after UV exposure varied by less than 6% and 5%, respectively. However, the strength of the respirator layers decreased significantly at the highest dose. This decrease was greater than 90% for the 710 and 950 J/cm^2^ doses (10).

Regarding respirator degradation, we know that polypropylene degrades with UV exposure. Polypropylene has high UV absorbance with low spectral variation under 400 nm, whereas polyester exhibits a significant peak in absorbance under 245 nm (11). Little research has been conducted regarding UV-C degradation of polypropylene. An article written for UV Solutions Magazine outlined the effect of UV on polymers. The article explained that UV damages polymers through chain scission photolysis. This observation means that the long polymer chains are broken down into smaller chains, reducing the strength of the material (12).

An indirect means of UV degradation is a result of radical formation. Radicals are highly reactive and degrade surrounding molecules by scission as well. As UV breaks down the molecules, the polymer surface is oxidized, furthering the damage to the material's structural integrity (12).

Further research is needed to determine if UVC damages the ability of polypropylene to retain viral particles. It is plausible that the true effects of UV are not adequately quantified when particles are used for experimentation instead of the microbial data. The respirators retain microbial surrogates due to electrostatic properties on the inside the respirator. The particles during testing may not share the same electrostatic properties as microbes. Therefore, the true interactions are not adequately measured. More research is needed to confirm that at 2,000 mJ/cm^2^ the electrostatic properties of the respirator begin to break down and release the microbial surrogates that were once retained in the respirator.

### Conclusions.

This research was spurred by increased interest in UV disinfection due to the COVID-19 pandemic. The major conclusions are listed below.

- For both MS-2 and E. coli, the data exhibited significant variation. This variation is due to the microbial enumeration techniques (plaque assays and IDEXX methods) and the variation between respirators. The efficacy of UV disinfection varies significantly between respirator types and models due to the differences in layers and materials. In addition, respirators of the same model type can behave differently.
- The respirator retention of viral and bacterial cells varied significantly between respirator types. This likely accounts for some variation in the UV dose-response curves. This variation is likely a result of the electrostatic interaction between the viral particle or bacterial cell in the middle layer of the respirator. The respirator with the most dramatic variation between MS-2 and E. coli retention was N95 8210.
- The dose-response curves for MS-2 and E. coli peaked at a UV dose of 1500 mJ/cm^2^ for all respirator types tested at that dose. The log inactivation values decreased at and above 2,000 mJ/cm^2^, this is thought to be due to degradation of the polypropylene material at these high UV doses. The polypropylene layer of the respirator provides the electrostatic charges that trap the viral particles and bacterial cells. It is predicted that the polypropylene layer begins to degrade at UV doses higher than 2,000 mJ/cm^2^, thus losing some of the electrostatic charges, allowing the surrogates to essentially wash out of the respirator when rinsed with solution.
- The respirator coupon experiments supported the hypothesis that UV doses higher than 2,000 mJ/cm^2^ have a degrading effect on the layers of the respirators. The irradiance values decreased interiorly, dropping in value between each layer. However, some of the layers that received the higher UV doses displayed a reduced ability to retain viral particles.

### Recommendations.

More studies must be conducted on the degradation of FFR materials using microbial surrogates. Fig. 1, 2, and 5 in this report suggested effects of degradation were seen at UV doses at and above 2,000 mJ/cm^2^. Although when the layers of the FFR were taken apart, the effects were not as exaggerated. More research must be done to determine if experiments conducted on FFR coupons are representative for full FFRs. In addition, research findings strongly suggest that the impacts of UV-C treatment on FFR material degradation should be confirmed using microbial surrogates in addition to abiotic particles.

## MATERIALS AND METHODS

### Respirators used for experimentation.

KN95-Purism is comprised of 5 layers. The layer that is furthest from the user's face (layer 1) is comprised of polypropylene spun-bonded nonwoven fabric. Layers 2 and 3 are comprised of polypropylene melt-blown nonwoven fabric. Layer 4 is comprised of polyethylene-polyethylene terephthalate air-blown nonwoven fabric. Finally, layer 5 is made of polypropylene spun-bound nonwoven fabric (13). Further information about KN95-Anboruo was not available.

The 3M N95 face respirators have 3 layers, referred to as the filter, shell, and cover-web. The filter of the respirators is comprised of polypropylene and the shells are made of polyester. The filter is made of advanced electrostatic media to trap viral and bacterial particles in the center of the face respirator. The N95 8210 cover-web is comprised of polyester, whereas the N95 8210-Plus cover-web is comprised of polypropylene (14).

### Experimentation.

**(i) UV device and optics.** A commercially available UV device (Lumin, manufactured by 3B medical), being tested by frontline workers was purchased and used for this research. An irradiance map was created using the data from an ILT770 research grade radiometer and modeled in MATLAB. The MATLAB model predicted contour lines of irradiance to define the UV optics of the device. The Lumin emits UV from the top of the device, where it has two lamps. The irradiance points were measured using a 13*13*13 data point grid in the x, y and z directions. The irradiance map and contact time was then used to determine the UV dose the respirators received at each location inside the UV Lumin device. More information regarding how UV dose on the respirators was calculated can be found in “UV Optics for Disinfecting Filtering Facepiece Respirators,” Bernardy et al. 2022.

**(ii) Surrogates.** This research used two organisms, Escherichia coli and Male Specific-2 Bacteriophage to create UV dose response curves. These surrogates have had widespread use in UV effectiveness and device validation studies based on EPA Disinfection Guidance Manual protocols (EPA, 2006). In addition, these organisms bracket the UV dose response of SARS-COV-2 allowing effective mathematical interpolation of results. Choosing E. coli in initial experiments also provided a relatively easy to assay, well-researched, bacteria for the experimental team to practice with while perfecting protocols and provided data to expand the database for all researchers examining the efficacy of UV devices to disinfect bacteria on PPE. MS-2 bacteriophage was selected as it was identified as being an adequate surrogate to SARs-CoV-2 for inactivation studies. These findings were based on previous research that demonstrated that SARs-CoV-1 and MS-2 bacteriophage displayed similar UV dose responses. There is limited data on acceptable surrogates for SARs-CoV-2 and the inactivation of SARs-CoV-1 and SARs-Cov-2 were expected to be similar, as they are both nonsegmented, enveloped, single-stranded, RNA viruses (15).

In addition, the body of knowledge is vast regarding water UV disinfection data for E. coli and MS-2 bacteriophage. The behavior of these surrogates has been studied by many researchers; therefore, we can confidently make comparisons between the dose response behavior observed with water data versus with FFRs (16).

The titer of the E. coli stocks was determined by assay 24 h prior to the scheduled experiment. Once the stock titer was confirmed,1 to 10mL of the surrogate of interest was added to990 to 999mL of Phosphate Buffer Solution (PBS). The PBS was created in reverse osmosis (RO) water and was autoclaved 24 h before experimentation to reduce potential contamination.

**(iii) Respirators.** Four respirator types (2 N95s and 2 KN95s) were exposed to UV doses and the inactivation of E. coli and MS-2 bacteriophage was measured. The respirator types that were used are referred to in the results section as KN95-Anboruo, KN95-Purism, and N95 8210, and N95 8210-Plus (Table 1).

**TABLE 1:** Manufacturer information for each respirator used during experimentation

Respirator type	Manufacturer
KN95A	Anboruo KN95. Manufactured at Yongkang Jinyang Plastic Products Co., LTD., in Zhejiang China.
KN95-Purism	Purism Daddy’s Choice KN95 Protective Face Mask. Manufactured at Shandong Daddy’s Choice Health Science and Technology Co., LTD., in Shandong Province China.
N95 8210	3M Particulate Respirator N95 8210. Manufactured in the USA with globally source materials.
N95 8210-Plus	3M Particulate Respirator N95 8210-Plus. Manufactured in the USA with globally source materials.

**(iv) Experimental procedure.** 8 mL of the surrogate in PBS solution was then pipetted into the reservoir of a nebulizer device (Pari-Vios Pro). The surrogates were aerosolized onto the FFRs using the nebulizer that produced a mean droplet size of 5 μm. As the nebulizer was intended for medical use, the discharge mouthpiece was modified with an Eppendorf 5,000 μL pipette to increase the velocity of vapor discharge and direct it in one centralized direction.

**FIG 8:**
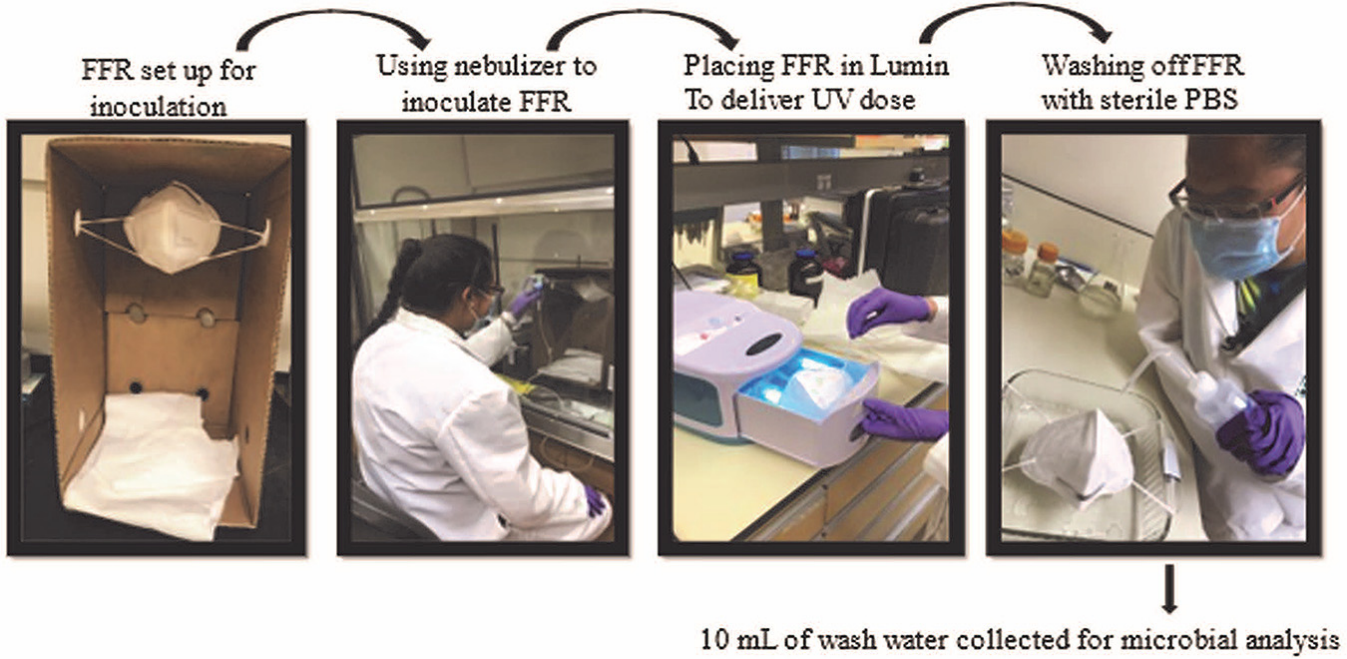
A schematic of the procedure followed for each experiment.

Aerosol application to the FFRs required approximately 30 min to ensure complete coverage. The pipette tip attachment was held ~0.5 to 1 in. away from the surface of the FFR and was applied in a slow sweeping motion to the outside the respirator. Three FFRs were tested per experimental day. One control and two trials were conducted at the specified UV dose. After the FFR was inoculated with the surrogate it was weighed, followed by a 15-minute dry time, weighed again, and placed into the Lumin for the specified exposure time. After being dosed in the Lumin, the FFR was immediately washed off with 500 mL of sterile PBS using a spray bottle with a narrow nozzle. The nozzle created a steady stream of PBS and was applied to the entire outer layer of the respirator. The PBS solution extracted the surrogates by both running off the outer layer and soaking through the FFR and dripping into the collection beaker. The control FFR was placed inside the UV device for the same amount of time as the trial respirators, but in the case of the control, the UV lights were not turned on. The control experiments were designed to determine the losses in the experiment due to surrogate retention on the FFRs and identify the extent of microbial variation between experiments. Results of the control FFR experiments were pooled to estimate the standard deviation of the experimental results and displayed in the figures as error bars. These data are provided in supplemental information. A schematic of the procedure followed for each experiment is displayed in Fig. 8.

For E. coli experiments, a 10 mL sample was taken of the PBS in the glass wash container and was serially diluted 7 times to reach a measurable range of approximately 1,200 organisms per 100 mL using the 97-well IDEXX Quantitray/2,000 MPN method. Supplementary data (Table S1 and Fig. S3) is provided which demonstrates the efficacy of this IDEXX method with midrange dilutions for samples containing high E. coli titers compared to plate count methods. The IDEXX trays were then placed in a 38-degree Celsius incubator for 28 h. The most probable number (MPN) of E. coli colonies per 100 mL of sample was determined from the IDEXX enumeration tables. The UV log inactivation value was determined by comparing the remaining viable bacterial cells to the control results. These data were adjusted to account for control experiments ensuring comparisons, results and conclusions were based on the more conservative and sensitive parameter of UV inactivation rather than the combination of retention on the FFR and UV inactivation.

For MS-2 experiments, two 30 mL samples of PBS from the glass wash container were taken from the control and each trial. The remaining viable MS-2 was quantified by using the double agar overlay method for determining phage plaques. From these data, the concentration of MS-2 could be determined. The UV log inactivation value was determined by comparing the remaining viable viral particles to the control results.

**(v) Degradation experiments.** Follow up experiments were conducted on KN95-Purism respirators to determine if doses above 2,000 mJ/cm^2^ caused material degradation which would be responsible for the respirators reduced ability to retain viral particles. These respirators were exposed to this UV dose prior to being inoculated with MS-2. Then the experiments followed the same procedures as above and comparisons regarding the ability of viral retention were made between the experimental respirators and the controls.

The research team also explored the effects of irradiance penetration through respirator layers. KN95-Purism and N95 8210 respirators were cut into coupons measuring approximately 5 cm by 5 cm in size. A collimated beam device was used to provide a more uniform dose to the small coupons. Therefore, the ILT1770 radiometer was utilized to measure the irradiance between each layer of the respirators. The experiments were then followed up with viral retention experiments on the coupons. The coupons were inoculated with 0.8 mL of MS-2 solution and dosed with UV at doses that mimicked the doses the received when the outer portion of the respirator received the dose at which degradation was thought to occur (2,000 mJ/cm^2^).

Please see https://doi.org/10.34051/d/2022.1 for raw data.
