# Supplementary material for: Effects of UV-C Disinfection on N95 and KN95 Filtering Facepiece Respirator Reuse
Source: Appl Environ Microbiol. 2022 Sep 21;88(19):e01221-22. doi: 10.1128/aem.01221-22 (PMC9552602; doi:10.1128/aem.01221-22)
Supplement: Supplemental file 1 — Supplemental material. Download aem.01221-22-s0001.pdf, PDF file, 0.3 MB [file aem.01221-22-s0001.pdf]

Effects of UV-C Disinfection on N95 and KN95 Filtering Facepiece Respirator Reuse

SUPPLEMENTAL INFORMATION:

Oneway Analysis of Inactivation By Mask Type

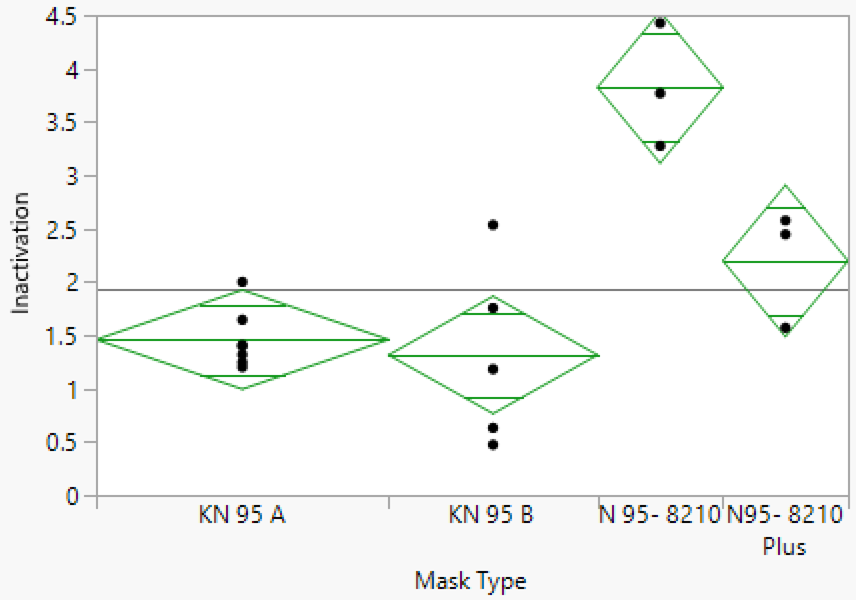

Oneway Anova

Summary of Fit

Rsquare	0.757822
Adj Rsquare	0.705927
Root Mean Square Error	0.573463
Mean of Response	1.936853
Observations (or Sum Wgts)	18

Analysis of Variance

Source	DF	Sum of Squares	Mean Square	F Ratio	Prob > F
Mask Type	3	14.406932	4.80231	14.6029	0.0001*
Error	14	4.604030	0.32886		
C. Total	17	19.010963			

Means for Oneway Anova

Level	Number	Mean	Std Error	Lower 95%	Upper 95%
KN 95 A	7	1.45987	0.21675	0.9950	1.9247
KN 95 B	5	1.31586	0.25646	0.7658	1.8659
N 95- 8210	3	3.82381	0.33109	3.1137	4.5339
N95- 8210 Plus	3	2.19784	0.33109	1.4877	2.9080

Std Error uses a pooled estimate of error variance

Figure S1 displays a statistical output from JMP for E. coli data. The output determines that there is statistical significance in the variation in mask retention between the 4 models used for experimentation.

Figure S1 displays an Analysis of Variance (ANOVA) on *E. coli* data created using the statistical software JMP. The *E. coli* retention values from Figure 5 were inputted into JMP and confidence intervals around the data points for each respirator type were created. The objective of creating an ANOVA table is to determine if the difference in mask retention of *E. coli* is significantly different. An F ratio above 1 indicates statistical difference, therefore the F ratio in the ANOVA table shown above (14.6) indicates that the difference in retention of *E. coli* between mask types is statistically different. In addition, it can be determined that the KN95 B (Purism) had the lowest retention and N95- 8210 the highest with values of 1.3 and 3.8 log respectively.

Oneway Analysis of Inactivation By Mask Type

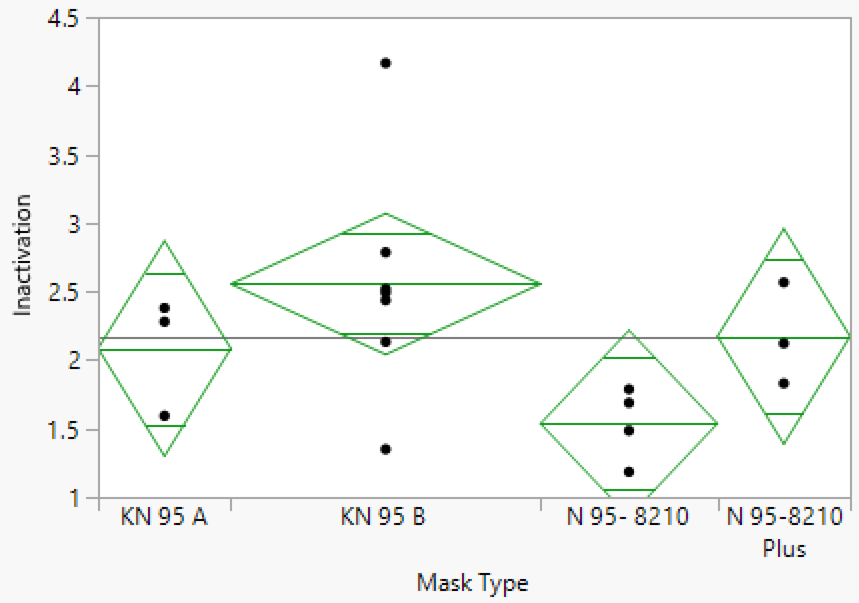

Oneway Anova

Summary of Fit

Rsquare	0.340529
Adj Rsquare	0.188343
Root Mean Square Error	0.629507
Mean of Response	2.165247
Observations (or Sum Wgts)	17

Analysis of Variance

Source	DF	Sum of Squares	Mean Square	F Ratio	Prob > F
Mask Type	3	2.6601254	0.886708	2.2376	0.1324
Error	13	5.1516213	0.396279		
C. Total	16	7.8117467			

Means for Oneway Anova

Level	Number	Mean	Std Error	Lower 95%	Upper 95%
KN 95 A	3	2.08499	0.36345	1.2998	2.8702
KN 95 B	7	2.55500	0.23793	2.0410	3.0690
N 95- 8210	4	1.53730	0.31475	0.8573	2.2173
N 95-8210 Plus	3	2.17335	0.36345	1.3882	2.9585

Std Error uses a pooled estimate of error variance

Figure S2 displays a statistical output from JMP for MS-2 bacteriophage data. The output determines that there is statistical variation in mask retention between the 4 models used for experimentation.

Figure S2 displays an ANOVA table for MS-2 data created in JMP. The F ratio (2.24) indicates that there is variation in viral retention between the four mask types used for experimentation. The table reveals that the N95 8210 had the lowest retention and KN95 B (Purism) had the highest, being 1.5 and 2.6 log respectively. These findings are the opposite of the findings from Figure S1, which is likely due to differences in the electrostatic interactions of the surrogates.

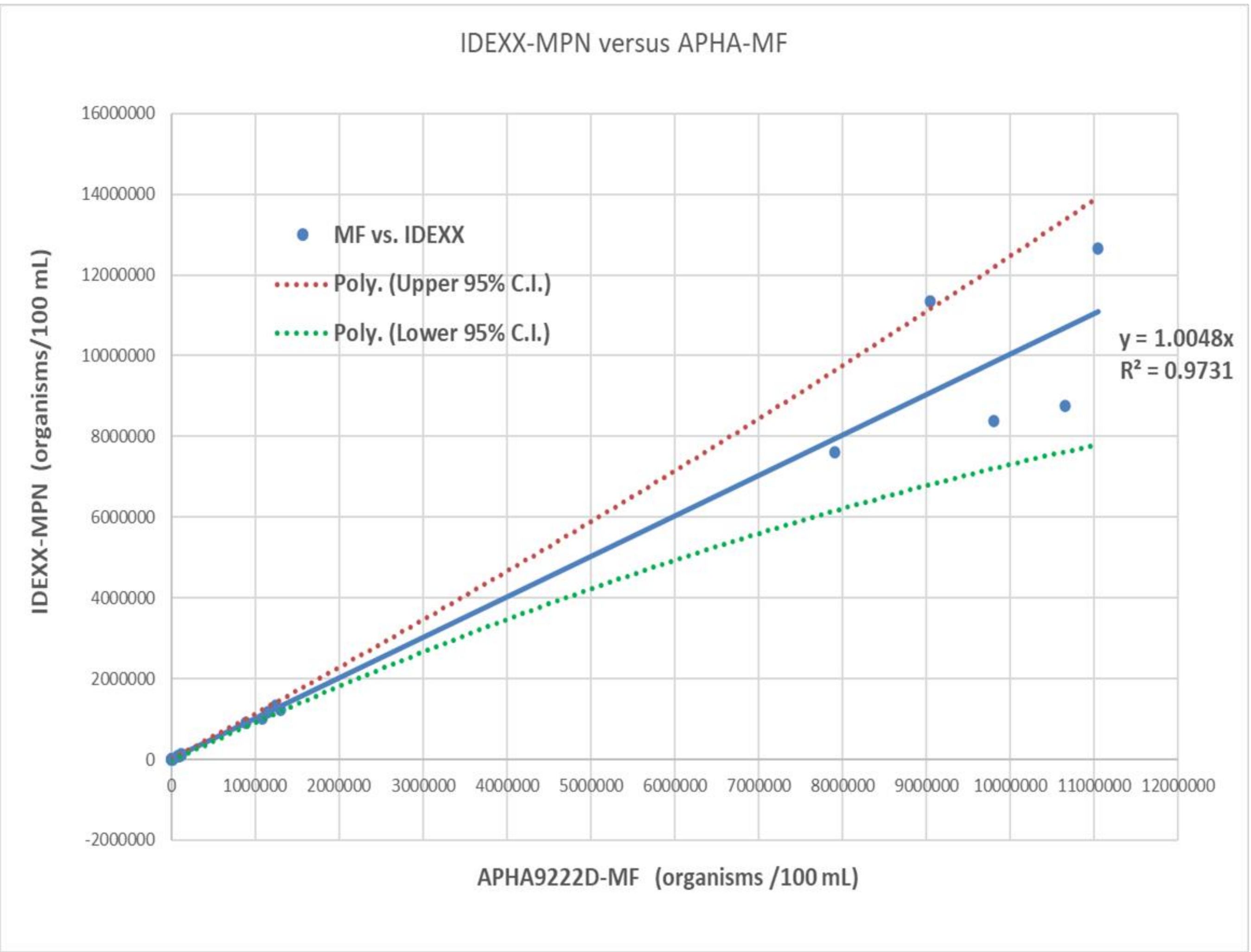

Figure S3 displays the graph created by the DeRISK QAPP data set. The data was collected by Kane-Malley in 2016 on MF versus IDEXX E.coli data.

These data display the method QAPP Results for IDEXX-Quantitray 2000 MPN Results versus APHA Method 9222D Membrane Filtration Method (Standard Methods for the Analysis of Water and Wastewater, 1995) The figure displays a strong correlation between the *E. coli* counting and IDEXX methods, with a high R^2 value of 0.9731. The raw data is shown below.

Oneway Analysis of Log Loss By Surrogate

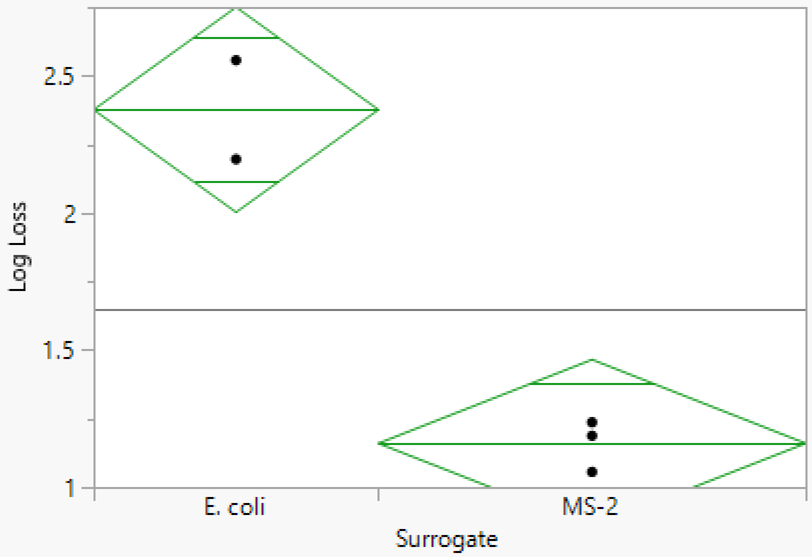

Oneway Anova

Summary of Fit

Rsquare	0.955485
Adj Rsquare	0.940647
Root Mean Square Error	0.166021
Mean of Response	1.6468
Observations (or Sum Wgts)	5

Pooled t Test

MS-2-E. coli

Assuming equal variances

Difference	-1.2162	t Ratio	-8.02454
Std Err Dif	0.1516	DF	3
Upper CL Dif	-0.7338	Prob > |t|	0.0040*
Lower CL Dif	-1.6985	Prob > t	0.9980
Confidence	0.95	Prob < t	0.0020*

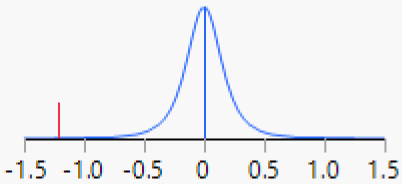

Analysis of Variance

Source	DF	Sum of Squares	Mean Square	F Ratio	Prob > F
Surrogate	1	1.7748736	1.77487	64.3932	0.0040*
Error	3	0.0826892	0.02756		
C. Total	4	1.8575628			

Means for Oneway Anova

Level	Number	Mean	Std Error	Lower 95%	Upper 95%
E. coli	2	2.37650	0.11739	2.0029	2.7501
MS-2	3	1.16033	0.09585	0.8553	1.4654

Std Error uses a pooled estimate of error variance

Figure S4 displays an ANOVA table created for the log loss of the surrogates when nebulized.

These data provide sufficient evidence that the log loss of E. coli through the nebulizer is significantly higher than the log lsos of MS-2 bacteriophage through the nebulizer. This can be seen by the visual variation in the location of the green mean diamonds and the very high F ratio (64.4), indicating a significant variation.

EPA NCISS - DeRISK QAPP Data Set

5. E. coli - UV Performance Data - response to questions on IDEXX Q2000 Method

8/2/2016

Kane-Malley

Test Level	Nominal(1) E. coli (# /100 mL)	APHA9222D (2) MF - E. coli (# /100 mL)	MF S.D.	MF 95% C.I.	MF Upper 95% C.I.	MF Lower 95% C.I.	IDEXX Q2000 (3) E. coli (# / 100mL)	IDEXX S.D.	IDEXX 95% C.I.	IDEXX Upper 95% C.I.	IDEXX Lower 95% C.I.
1	100	72			92.296612	51.7034	71			95.8270716	46.17293
		96			116.29661	75.7034	75			99.8270716	50.17293
		80			100.29661	59.7034	70			94.8270716	45.17293
		66			86.296612	45.7034	67			91.8270716	42.17293
		105	16.3463	20.2966	125.29661	84.7034	115	19.995	24.82707	139.827072	90.17293
2	1000	1210			1358.1951	1061.8	1274			1500.70301	1047.297
		980			1128.1951	831.805	905			1131.70301	678.297
		895			1043.1951	746.805	833			1059.70301	606.297
		1100			1248.1951	951.805	1172			1398.70301	945.297
		1050	119.352	148.195	1198.1951	901.805	1063	182.58	226.703	1289.70301	836.297
3	10,000	9760			10917.062	8602.94	9900			11364.0709	8435.929
		11,500			12657.062	10342.9	10190			11654.0709	8725.929
		9950			11107.062	8792.94	11720			13184.0709	10255.93
		8950			10107.062	7792.94	8550			10014.0709	7085.929
		9750	931.864	1157.06	10907.062	8592.94	9310	1179.12	1464.071	10774.0709	7845.929
4	100,000	99800			121370.93	78229.1	80900			105886.377	55913.62
		89000			110570.93	67429.1	90500			115486.377	65513.62
		115000			136570.93	93429.1	113400			138386.377	88413.62
		78500			100070.93	56929.1	88200			113186.377	63213.62
		120000	17372.6	21570.9	141570.93	98429.1	129100	20123.3	24986.38	154086.377	104113.6
5	1,000,000	1250000			1451985.2	1048015	1331000			1542118.89	1119881
		1300000			1501985.2	1098015	1236000			1447118.89	1024881
		1085000			1286985.2	883015	1022000			1233118.89	810881.1
		1155000			1356985.2	953015	1172000			1383118.89	960881.1
		885000	162673	201985	1086985.2	683015	905000	170029	211118.9	1116118.89	693881.1
6	10,000,000	9050000			10618030	7481970	11340000			14000373.1	8679627
		7905000			9473030.4	6336970	7620000			10280373.1	4959627
		11050000			12618030	9481970	12660000			15320373.1	9999627
		10650000			12218030	9081970	8760000			11420373.1	6099627
		9800000	1262846	1568030	11368030	8231970	8390000	2142587	2660373	11050373.1	5729627
Sterile Controls											
1		0					<1				
2		0					<1				
3		1					<1				
4		0					<1				
5		0					<1				
Spiked Positive Controls	Target										
1	1	<1					<1				
2	10	8					12				
3	500000	415000					408000				
4	5000000	5750000					5350000				
5	11000000	10500000					10710000				

Notes:

1. Estimated by calculation from Stock E. coli 25922(Tm) ATCC
2. APHA Standard Methods for the Analysis of Water and Waste Water Method 9222D - MF (20 to 200 counts per plate accepted)
3. IDEXX™ Quantitray 2000 System - MPN technique - Table value round up to next whole integer

(Dilutions used for the Q2000 trays of 100 mL sample producing a value in the 40x40 wells on the MPN table = 203 organisms/100 mL or less)

Table S1 displays the DeRISK QAPP data set. The data was collected by Kane-Malley in 2016 on MF versus IDEXX E.coli data.

These data were collected as part of an EPA grant and suggest a strong correlation between standard plating techniques and IDEXX methods. A figure of the data is shown above.

Log loss (retention) of *E. coli* control runs

	KN95-Anboruo	KN95-Purism	N95 8210-Plus	N95 8210
	1.202	0.632	2.576	3.276
	1.319	0.474	1.571	4.427
	1.645	2.535	2.447	3.769
	1.408	1.182		
	2.001	1.756		
	1.246			
	1.400			
**Average**	**1.460**	**1.316**	**2.198**	**3.824**
**St. Deviation**	**0.258**	**0.758**	**0.446**	**0.472**

Table S2 displays the retention of the E. coli cells in the four FFRs used for experimentation.

These data were collected from the control runs conducted for each experiment, where the FFRs where not exposed to UV. The standard deviation for each FFR type was used to create the error bars for the dose response data shown in Figure 2. In addition, these data points were used in the ANOVA tables shown above in Figure S1.

Log loss (retention) of MS-2 bacteriophage control runs

	KN95-Anboruo	KN95-Purism	N95 8210-Plus	N95 8210
	2.281	2.420	2.122	1.788
	1.594	1.350	1.831	1.689
	2.380	2.134	2.567	1.186
		2.520		1.487
		2.787		
		2.437		
**Average**	**2.085**	**2.286**	**2.173**	**1.537**
**St. Deviation**	**0.350**	**0.460**	**0.303**	**0.230**

Table S3 displays the retention of the MS-2 bacteriophage particles in the four FFRs used for experimentation.

These data were collected from the control runs conducted for each experiment, where the FFRs where not exposed to UV. The standard deviation for each FFR type was used to create the error bars for the dose response data shown in Figure 3. In addition, these data points were used in the ANOVA table displayed above in Figure S2.
